# Negatively Marked Elimination-Format Multiple-Choice Questions Are Associated with High Cognitive Load and Poor Student Experience Compared to Single Best Answer

**DOI:** 10.1007/s40670-025-02318-7

**Published:** 2025-02-17

**Authors:** Philip M. Newton, Katherine H. Furby, Jude Campbell, Atharva Salvi, Gabriella Santiago, Michael Chau

**Affiliations:** Swansea University Medical School, Swansea, SA2 8PP UK

**Keywords:** Multiple-choice questions, Single best answer, Elimination testing, Cognitive load, Testing effect

## Abstract

Multiple-choice questions (MCQs) are a common form of assessment in medical science education. The traditional MCQ format involves students picking a single best answer (SBA) from four or five options. There are concerns about the ability of SBA formats to reward partial knowledge and their susceptibility to guessing. An alternative to SBA is elimination testing (ET), wherein students eliminate all the *incorrect* answer options, with negative marking to deter guessing. Cognitive load theory (CLT) is an approach to education that prioritises strategies to minimise the amount of unnecessary ‘load’ placed upon working memory. The cognitive load imposed by assessment design has received little attention. We evaluated the cognitive load of SBA and ET MCQ formats, using an online participant pool and a survey of students at a UK Medical School. We found that partial knowledge was rewarded with the ET format. However, students strongly preferred the SBA format and reported both that it was easier and imposed a lower cognitive load. Removing negative marking reduced the cognitive load of ET style questions and improved the student experience, but the improvement was insufficient to change student preference for SBA questions.

## Introduction

Examinations based on multiple-choice questions (MCQs) are a very common form of assessment in medical science education and licensing. There are well-established principles for the effective construction of effective MCQs [1], and, if written appropriately, they can be used to assess ‘higher order’ learning [2]. Assessments based on MCQs can deliver an objective assessment, with broad coverage of the curriculum, that is inclusive of learners with specific learning needs [3, 4].

The traditional MCQ format involves students picking a single best answer (SBA) from a list of four or five answer options. Students are awarded one mark for selecting the correct answer and no marks for selecting the incorrect answer. Despite their widespread use, there are some well-established potential limitations with this SBA format. Students can theoretically achieve a certain percentage of ‘correct’ answers through simple guesswork [5], and this effect can be enhanced by a cueing effect in poorly designed questions where the correct answer is signposted [6]. This cueing effect is more common with difficult questions [7]. The SBA format also does not reward partial knowledge, for example, where a student is unsure of the precise correct answer but is sure that one or more of the answer options is incorrect.

Alternative MCQ formats have been proposed to address some of these concerns [5]. One of these is the elimination-type (ET) format, a format where students are asked to eliminate every incorrect answer they can identify. The more incorrect answers they eliminate, the more marks they get. Thus, students can get credit for partial knowledge by only eliminating those answer options that they know to be incorrect, even where they are not fully confident in their choice of correct answer and so allowing for a more granular assessment of learning [8].

However, the pure ET format does not eliminate guesswork and may make it worse. For example, in a five-option MCQ, there is the chance to gain a maximum of four marks by eliminating all four incorrect answer options. If the learner picks four at random, they will always pick at least three incorrect options and so obtain a minimum of three marks. A common strategy to minimise the use of this random guessing is to deploy negative marking, where students lose marks for eliminating the correct answer, penalising incorrect knowledge and improving test reliability [9].

However, negative marking has complications of its own, not least that students do not like it [10], and prior research has shown that students prefer traditional SBA questions to negatively marked ET [11]. Also, there is some controversy over whether negative marking actually reduces guesswork [12, 13], although this may be in part because of misunderstandings about how and when to use negative marking [14]. One proposed solution to this uncertainty is simply to negatively mark both the SBA and ET formats; a 2013 study of low-scoring formative assessments found that this approach did result in students preferring the ET format [15] although it retains the negative marking and associated challenges.

The current study sought to determine whether negative marking is a feature which students dislike about ET format MCQs, and whether there are other features of ET format MCQs which may explain why students dislike them, in particular the cognitive load associated with them.

Cognitive load theory (CLT) is an established approach to instructional design but has not been extensively studied in assessment. CLT is focused on the limitations of our working memory which has an extremely limited capacity for information processing and storage and yet is fundamental to human learning [16]. The ‘load’ in cognitive load theory refers simply to the load placed on this limited resource of working memory. CLT proposes that ‘instructional design decisions should be informed by the architecture of. the human cognitive system’, of which this limited working memory capacity is perhaps the most significant [17].

It has been proposed that there are two or three different types of cognitive load, each distinct from the other. Perhaps, the most important for instructional design is extraneous load which is, simply, any feature of the instructional material which takes up some of the limited space in human working memory but without contributing to learning. For example, decorative features of instructional materials can make those materials harder to remember, but not because of the complexity of the underlying *content* of the materials, but simply through poor design of the presentation [18]. By reducing extraneous load, educators can increase the efficacy of instructional materials. Intrinsic cognitive load is essentially the academic difficulty of a topic, for example, calculus has a higher intrinsic load than basic arithmetic [19]. Cognitive load can be measured in multiple ways with the most common method being subjective self-report, using single or multi-dimensional scales. The original Paas nine-point single scale continues to be very popular when measuring cognitive load in studies of medical science education [20, 21].

Cognitive load in instructional design has been the subject of several reviews and textbooks [17, 22–25], but there has been very little research into the cognitive load of different assessment formats and the impact of manipulating cognitive load on the student experience of different assessment formats. It seems important to determine the cognitive load of assessment materials; if the design of assessment materials increases extraneous cognitive load, then this is going to make the assessment item harder to answer but in a way that is unrelated to the underlying learning being assessed. It has also been proposed that a heavy assessment burden will lead to high overall cognitive load and thus poor performance [26] or increase the likelihood that students will engage in academic misconduct [27], but there are limited empirical data to support these hypotheses.

The ET MCQ format requires students to consider all answer options, especially where they are uncertain of which one is correct. We hypothesised that this will impose higher cognitive load than the SBA format and that this load will be exacerbated by the use of negative marking. We conducted three studies to test and develop our ideas. In Study 1, we used an online participant pool to directly compare the cognitive load and student preferences for the SBA vs the negatively marked ET format. In Study 2, we surveyed the views of students who had experience of both SBA and negatively marked ET formats for summative assessment at a UK university. In Study 3, we repeated Study 1 but using neutrally marked ET questions. The use of an online participant pool allowed us to evaluate student preferences in a completely independent manner, free from the influence, conscious or unconscious, of educators and their institutions.

## Methods

### Participants (Studies 1 and 3)

Participants were recruited from the online pool www.prolific.com. Participants were restricted to those currently studying a science topic at undergraduate degree level. The specific degrees allowed were Medicine, Biochemistry, Dentistry, Biomedical Sciences, Genetics, Pharmacology, Biological Sciences, Veterinary Science, Physics, Engineering, Nursing, Chemistry, Biology, Earth Sciences, Health and Medicine and Material Sciences. Participants were paid the rate recommended by Prolific, which at the time was GBP 2.40 for satisfactory completion of the study. Sixty-five participants were recruited for Study 1. (The original intention was to recruit 2 × 32 but due to a technical error an additional participant was recruited to one of the groups.) Two ‘attention check’ questions [28] were included where the only appropriate answer option was ‘I am paying attention to this study’. One participant from Study 1 answered both attention check questions incorrectly, and so their data were removed, resulting in an ‘*N*’ of 33 participants answering questions in a certain format, and *N* = 31 for others. Fifty new participants were recruited for Study 3, none of whom failed both attention checks.

### Study Instrument Structure (Studies 1 and 3)

The study instrument was constructed in Qualtrics™ and included 40 general science MCQs aimed at a level that year 1 UK STEM undergraduate students could reasonably answer, based on A-level and GCSE (UK schools exams) science revision questions [29–33]. The full question set is shown in Appendix 1. Each question was constructed in both SBA and ET format. The study instrument was piloted twice on Prolific for clarity and the overall level of difficulty of the questions. Following the first pilot, colour cues were added to highlight the question format being answered at any one time, along with additional instructions regarding the format of the questions. A second pilot was conducted with eight participants per group. No further changes were required, and so the second set of pilot data were included in the final analysis. The study settings on Prolific were configured to ensure that no participants could take the study twice.

#### Introduction

The purpose of the study was explained (see below). Participants were asked to refrain from consulting any external sources and to complete the survey to the very best of their ability. They were told that they would be paid for any satisfactory contribution made and that the submission of their answers indicated consent.

#### Instructions

The two different question formats were explained, along with the way in which they would have been scored if this was an actual test (SBA questions: one point for selecting the correct answer and no points for selecting the incorrect answer; ET format: one point for each of the four incorrect answer options eliminated, but − 4 points for eliminating the correct answer (Study 1; negative marking) or zero points if they eliminated the correct answer (Study 3; neutral marking)), regardless of how many incorrect options had been eliminated. The negative mark of − 4 in Study 1 was used to match the real-world experience of the students using ET formats for summative assessment in Study 2.

#### Practice Question

The explanation was then followed by three practice questions in each format. All practise questions were easy (for example, ‘what colour is the sky’) so the participants could focus on the format rather than the content.

#### Study Questions

These were presented in four groups of ten. Two groups of participants were recruited in each study. Both groups received the same forty questions, in the same order. However, the first group (*N* = 31 participants in Study 1, *N* = 25 in Study 3) received questions 1–10 and 21–30 in ET format and questions 11–20 and 31–40 in SBA format. This formatting was reversed for the second group (*N* = 31 participants for Study 1, *N* = 25 for Study 3). Thus, all questions were tested in both formats, and the presentation of the formats was counterbalanced.

#### Cognitive Load

Participants were asked, in separate questions, to ‘rate the mental effort required to complete’ each of the question types using the Paas 9-point scale [34], where 1 is ‘very, very low mental effort’, and 9 is ‘very, very high mental effort’.

#### Student Experience

Participants were asked ‘of the two aforementioned questioning styles, which did you find easier’ and ‘of the two aforementioned questioning styles, which did you prefer’. In Study 3, participants were then asked additional questions to further explore their reasoning. This section was then branched so that participants who said they preferred SBA were asked different questions to those who said they preferred ET. These questions were used in Study 2 and were derived from the existing literature on the student experience of the different question types [35].

#### Clarity Check

Participants were asked whether (1) the format of the survey was clear and (2) the format of the questions was clear.

Finally, participants were asked if they had any further feedback. They were then debriefed and redirected to Prolific and paid.

### Study 2. Survey of Students for Whom Elimination MCQs Are Part of Their Summative Assessment

This survey was administered to students studying Medical Science degrees at a medical school in the UK (not medical students). These are a suite of 19 different undergraduate degrees, in which ET MCQs are used as part of a portfolio of summative assessment methods, and so students have experience of this format.

#### Survey Instrument Structure

The structure of the two question formats was explained, and participants were asked to take the example questions as in Studies 1 and 3. They were then asked to confirm that they had taken a multiple-choice exam, in either format and then in a separate question to confirm that they had taken an MCQ exam in the ET format. They were then asked which of the two formats they preferred, followed by four Likert scale questions designed to understand their reasons for their preference. These Likert questions were based upon prior literature which examined students preference for the two formats [35]. The Likert questions were specific to the preferred format (i.e. students that preferred SBA-type questions were asked four question about why they preferred that format). All students were then asked a free-text question ‘Is there anything else you would like to add about your preference (or lack thereof) towards exam styles?’.

#### Survey Distribution and Sample

We used a convenience sample. The survey was sent via email to all 827 enrolled students from UK FHEQ Level 3 to 6 (foundation undergraduate through to final year undergraduate). The email stated that the researchers ‘are running a very short survey on student experiences of certain types of exam questions. Your responses are entirely anonymous and the survey should take less than 5 min. The project has ethical approval from the SUMS RESC’.

#### Participants

One hundred eighteen students responded, with 107 completing the survey, giving a response rate of 13%. Of these, 90 answered yes to both confirmation questions (i.e. had sat MCQ-based exams, including in the ET format), and so data are presented from those students.

### Analysis

Data are reported as mean ± standard error. All datasets were tested for normal distribution using a Kolmogorov–Smirnov test [36]. Non-parametric tests were used where data were not normally distributed. The specific statistical tests used are identified in the relevant section of the results.

### Ethics

Ethical approval was given by the Swansea University Medical School Research Ethics Committee, code SUMS-RESC 2019–0060. All participants gave electronic written consent, and all data were anonymous.

## Results

### Study 1. Cognitive Load of Single Best Answer vs Negatively marked Elimination-Style Questions

#### Cognitive Load

**Fig. 1:**
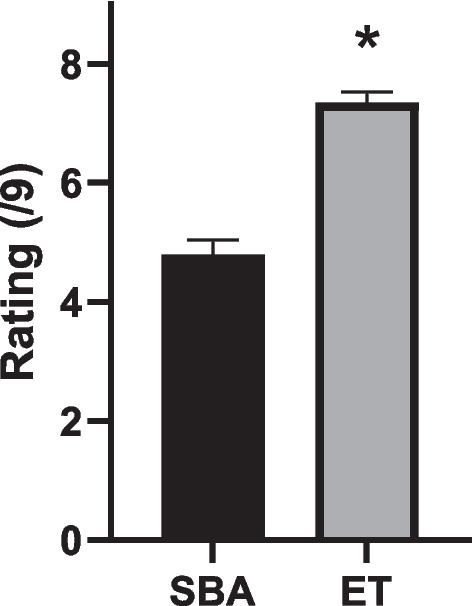
Negatively marked elimination format MCQs (ET) are associated with a higher self-reported cognitive load than traditional single best answer MCQs (SBA). *P < 0.05

A Wilcoxon matched-pairs signed-rank test was performed in order to compare the mean cognitive load of the two MCQ formats. The cognitive load assigned to SBA ranged from 1 to 9 on the Paas 9-point scale with a mean of 4.80 ± 0.244. The cognitive load assigned to Elimination-style ranged from 2 to 9 on the Paas 9-point scale with a mean of 7.34 ± 0.18. This difference was statistically significant according to a Wilcoxon matched-pairs signed-rank test (*W* = 1635, *P* < 0.0001) (Fig. 1).

#### Preferences

One participant did not answer these questions, meaning *N* = 63. A substantial majority of participants stated that they preferred the SBA questions (78%) and found them easier (83%) (Table 1).

**Table 1:** Participants in Study 1 prefer multiple-choice questions in the single best answer format and report finding them easier than negatively marked elimination-style format

	Easier	Preferred
Single best answer	52 (83%)	49 (78%)
No preference	7 (11%)	9 (14%)
Elimination-style	4 (6%)	5 (8%)

Data shown are given as participant frequencies with corresponding percentages in brackets (*N* = 63)

An analysis of individual participants showed that only 6/64 participants (9.4%) reported a lower cognitive load from the ET MCQs. Only 3/64 participants (4.7%) stated that they both preferred the ET format and found them easier. These three participants all belonged to the group of six who reported a lower cognitive load from the elimination-style.

#### Partial Credit

One of the proposed benefits of the ET format is that partial knowledge can be rewarded, whereas SBA MCQs can only reward full knowledge. We analysed by comparing the number of participants who received any credit for each version of the question and then calculating the average per format. These values were converted to percentages to correct for the slight difference in sample size. A paired *t*-test showed a significant difference between the two formats (*t* = 2.92, *P* < 0.0001), with an average of 64.6 ± 3.4% of participants gaining credit for the SBA format of a question vs. 70 ± 2.6% for the ET format. There was a strong and significant correlation between these two datasets when analysed by calculating a Pearson correlation coefficient (*r*_(38)_ = 0.82, *P* < 0.0001). A similar correlation was obtained when comparing the mean score per question (*r*_(38)_ = 0.84, *P* < 0.0001). These correlations indicate that certain questions were more difficult than others and that the format of the question did not significantly affect how difficult the question was to answer.

### Study 2. Student Experience Survey with Participants Who Have Experience of Both SBA and Negatively Marked ET MCQ Formats, in Summative Assessments

**Fig. 2:**
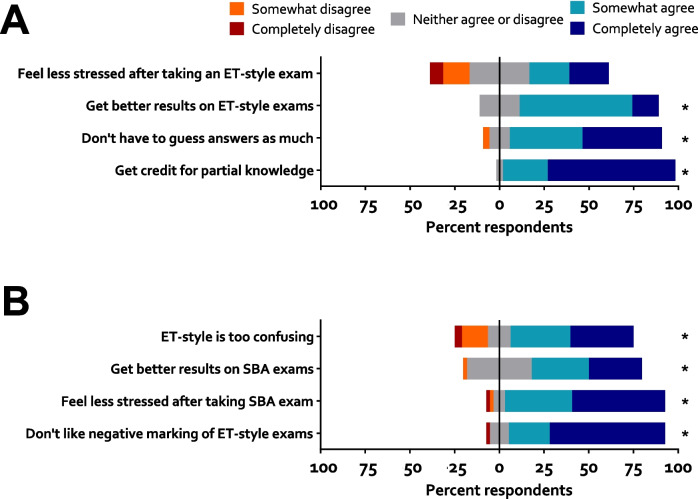
Reasons for students to prefer either **A** negatively marked elimination (*N* = 29) or **B** single best answer format (*N* = 48) multiple-choice questions. Answers were solicited from students who expressed a preference for that format, using a Likert scale. Distribution of response was analysed using a one-sample Wilcoxon signed-rank test with a hypothetical median of ‘3’ (neither agree or disagree). *P < 0.05

There was again a preference for the SBA format with 48 (53.3%) participants preferring it. Twenty-nine (32.2%) stated that they preferred the ET format. Thirteen (14.4%) reported no preference. Participants were then asked to rate their agreement with statements regarding their stated preference. To test whether agreement, or disagreement, was significant, the data from each question were converted to ordinal numbers from 1 (strongly disagree) to 5 (strongly agree). Each question was then analysed using a one-sample Wilcoxon test to determine whether the distribution of the data were significantly different from 3 (‘neutral’, the midpoint of the range). There was significant agreement (*P* < 0.05) with all questions except for a question asked of participants who preferred the ET-format. That question was ‘I feel less stressed after taking an elimination-style exam’ (*P* = 0.1467, *W* = 69). The distribution of responses to all questions is shown in Fig. 2.

#### Free Text Comments

Only 20 participants left comments in response to the question ‘Is there anything else you would like to add about your preference (or lack thereof) towards exam styles?’, and all were brief, and so a full thematic analysis was not possible. However, eight of the comments stated that negative marking was a reason to dislike ET style questions. Comments in favour of the SBA format indicated that they were simpler and familiar and took less time.

### Study 3. Cognitive Load of Single Best Answer vs. Neutrally Marked Elimination-Style Questions

To test whether the removal of negative marking would change student views of the ET format, we repeated Study 1, but without negative marking. Participants still preferred the SBA to the ET and found them easier, but the percentages were lower than in Study 1 (Table 2).

**Table 2:** Participants in Study 3 prefer multiple-choice questions in the single best answer (SBA) format and report finding them easier than neutrally marked elimination format

	Easier	Preferred
Single best answer	**33 (66%)**	**36 (72%)**
No preference	**7 (14%)**	**5 (10%)**
Elimination-style	**10 (20%)**	**9 (18%)**

Data shown are given as participant frequencies with corresponding percentages in brackets (*N* = 50)

A chi-squared test was used to determine whether the switch from negative to neutral marking affected student preference. This showed a significant difference for the question about whether participants found the SBA format easier *χ*^2^ (2, *N* = 113) = 12.98, *P* = 0.0015. The difference was not significant when asking about which format the participants preferred, although it was borderline χ^2^ (2, *N* = 113) = 4.753, *P* = 0.0929. These data show that the numbers who prefer the SBA test are significantly higher when the ET questions are negatively marked and show a trend for participants to find them easier as well.

#### Reduction in Cognitive Load Following the Removal of Negative Marking

The mean cognitive load score for the ET format reported by participants in Study 1 (with negative marking) was 7.34 ± 0.184. The mean cognitive load score for the ET format reported by participants in Study 3 (with neutral marking) was 6.68 ± 0.2329. The difference between these two was significant according to a Mann–Whitney *U* test (*P* = 0.024, *U* = 1215), with an effect size Cohen’s *d* = 0.42. There was no significant difference between the mean cognitive load reported for the SBA format in the two studies (*P* = 0.3363, *U* = 1433, Mean = 4.8 ± 0.2442 for Study 1, 5.14 ± 0.2983 for Study 3, *d* = 0.179). These data suggest that negative marking is associated with a higher cognitive load of ET format questions.

Again, a small number of participants (*N* = 10/50, 20%) reported a lower cognitive load from the ET MCQs. Five (10%) stated that they both preferred the ET format and found them easier. These participants all belonged to the group of ten who reported a lower cognitive load from the ET format.

#### Relationship Between Cognitive Load and Question Difficulty

To test whether there was a relationship between overall question difficult and self-reported cognitive load, we tested for a correlation between participants total score on the SBA questions and the self-reported cognitive load of the SBA questions. These data were not normally distributed, and so a Spearman correlation test was used, which did not indicate a significant difference (*P* = 0.195, *r* =  − 0.1223).

## Discussion

Our findings suggest that students prefer question formats that are easier to answer and have lower cognitive load and without negative marking, even when that format does not reward partial knowledge.

Specifically, the findings support our hypothesis that ET-format MCQs impose a higher cognitive load than SBA MCQs. We can also conclude that, despite participants preferring the SBA style of MCQ over the ET-style, they got more credit for the ET-style. Our findings also suggest that student preference for the SBA format is explained, in part, by the negative marking associated with the ET format and that this negative marking is associated with a higher cognitive load. However, a removal of the negative marking does not completely eliminate student preference for SBA or the higher cognitive load associated with the ET format.

A small number of participants across Studies 1 and 3 (8/114, 7%) reported that they both preferred the elimination-style and found them easier. These participants were all also in the small group (16/114, 14%) who reported lower cognitive load from the elimination style compared to the SBA format. These numbers are too low for any firm conclusions but they are, when considered as part of the analysis overall, supportive of a causal link between perceived cognitive load and question difficulty/preference. This subset of the data presents an interesting avenue for further work.

We can be reasonably confident that the academic difficulty of the questions was not meaningfully impacted by the question format; the numbers of students getting credit for any one question was correlated between formats. There also did not appear to be an overall relationship between participant performance and cognitive load. These findings suggest that the ET-format adds extraneous cognitive load, rather than increasing the intrinsic cognitive load of the assessment item. An analysis of the different types of load imposed by assessment formats, and the impact on retrieval practice, is the subject of future research using multidimensional instruments to measure the different types of cognitive load [37] and using objective measures of cognitive load alongside the common but self-reported Paas scale used here.

Regardless of an effect on learning or performance, it is also important to consider the result from a pragmatic perspective; pragmatism is an approach to research which prioritises the generation of findings that are practically useful for everyday practitioners [38]. It was for this reason that we collected participants subjective opinions about whether they preferred the different question formats. These subjective experience data are low on a traditional evaluation framework, but the student experience is fundamentally important to many aspects of higher education and is, rightly or wrongly, used as a measure of teaching quality and informs hiring/promotion decisions about academic staff [39, 40], despite the many methodological and conceptual challenges of student experience surveys [41]. Thus, our finding that a substantial majority of students dislike the ET format is an important consideration for any educator who is contemplating using them. Our finding that this dislike of the ET format persists even after the removal of negative marking suggests that, despite the reward of partial credit, educators may still suffer negative consequences themselves should they deploy the ET format.

Our data also suggest that there are additional features of the two formats which lead students to prefer SBA over ET. Participants in Study 2 reported feeling less stress after taking summative exams in the SBA format and perceived that they got better results, despite the additional credit achieved under the ET format. They also reported that the ET-style was ‘too confusing’, which itself may be related to cognitive load. Unpicking these features is also the subject of ongoing work.

It is possible that novelty might partially explain our results, although we did not test for it explicitly here; most students have experienced SBA-type questions at some point, whereas ET are less common, and so participants may have found ET harder/less preferable in part because they are not used to them. Familiarity can lead to preference over novelty in choice experiments [42], whereas novelty is an obvious source of increased cognitive load. All participants in Study 2 had experience with the ET format in summative assessments, and yet still preferred the SBA format, although the size of the difference appears to be lower. Also, even if novelty is part of the explanation then, from the pragmatic perspective, the results will still impact any educator who wishes to use the ET format with students; the only solution to this would be to make extensive, possibly exclusive use of the ET format in both formative and summative assessments, to reduce novelty and give students experience and comfort with the format. By adopting this strategy and thus making the ET format familiar, educators could reduce any novelty associated with them and so possibly reduce cognitive load. This could be a simple route to delivering on some of the obvious benefits of the ET format, in particular the rewarding of partial knowledge.

Perhaps most importantly, our findings indicate the importance of further examination of the effect of assessment design on cognitive load and the subsequent impacts on the psychometric performance of the assessment along with the student experience. Our findings suggest that assessment design can profoundly affect cognitive load and that this impairs the student experience.

## Data Availability

The datasets generated during and/or analysed during the current study are available from the corresponding author on reasonable request.

## Appendix

### STEM Questions. Correct answer is highlighted in yellow

1. Which of the following is a known disadvantage of using an Electron Microscope?
  A It has a low resolution
  B You cannot use live specimens
  C They can only allow a low level of magnification
  D The colours of the image are very bright
  E The samples must be wet
2. What is an isotope?
  A An atom that has more electrons than the standard for that element
  B An atom that has fewer protons than the standard for that element
  C A standard atom of a particular element
  D An atom that has a different number of neutrons than the standard for that element
  E An atom that has the same number of neutrons as it does protons
3. Where does photosynthesis take place?
  A Chlorophyll
  B Mitochondria
  C Nucleus
  D Cell Wall
  E Ribosomes
4. Which value is Avogadro’s constant?
  A 1.2 × 10⁷
  B 4.3 × 10^1^⁶
  C 6.022 × 10.^23^
  D 7.4 × 10⁸
  E 5.77 × 10^1^⁷
5. What are the nucleotide pairings in a strand of RNA?
  A C and T, A and G
  B A and T, G and C
  C U and C, G and T
  D T and U, C and G
  E A and U, G and C
6. Which of the following anatomical structures is not involved in digestion?
  A Appendix
  B Gall Bladder
  C Small Intestines
  D Pancreas
  E Salivary Glands
7. Which of the following is not a geometric arrangement of a molecule?
  A Trigonal Planar
  B Decahedral Pyramid
  C Seesaw
  D Trigonal Bipyramidal
  E Tetrahedral
8. Which of the following has the molecular formula, CH_3_COOH?
  A Ethanol
  B Propane
  C Propanoic Acid
  D Ethanoic Acid
  E Pentanol
9. Which of the following describes Newton’s Second Law of Motion?
  A P = fv
  B Q = mc∆T
  C F = ma
  D g = F/m
  E F = k∆L
10. Which of the following is not a moon of Jupiter?
  A Io
  B Ganymede
  C Europa
  D Titan
  E Callisto
11. Which of the following is the speed of light?
  A 74000 miles per second
  B 186000 miles per second
  C 896000 miles per second
  D 415000 miles per second
  E 675000 miles per second
12. What is the name of the solar system where Earth is located?
  A Calor
  B Sol
  C Pangea
  D Luna
  E Universo
13. Which of the following describes a solar system that is centred around the Sun?
  A Holocentric
  B Geocentric
  C Ptolemaic
  D Acrocentric
  E Heliocentric
14. Which of the following requires ATP to take place?
  A Active Transport
  B Diffusion
  C Osmosis
  D Facilitated Diffusion
  E Filtration
15. What is the water potential of pure water?
  A 7
  B 92
  C 100
  D 24
  E 0
16. What is another term for geographical speciation?
  A Allopatric
  B Geopatric
  C Mechanical
  D Heteropatric
  E Homopatric
17. By which method does DNA replicate?
  A Conservative
  B Dispersive
  C Complementary
  D Semi-conservative Replication
  E Binary fission
18. What is the definition of a nucleophile?
  A A substance that accepts electrons
  B A substance that accepts neutrons
  C A substance that donates electrons
  D A substance that is stable
  E A substance that donates protons
19. Which of the following describes a weak acid?
  A Partial dissociation in solution
  B No dissociation in solution
  C A substance that has an equal ratio of protons to electrons
  D Complete dissociation in solution
  E Partial association in solution
20. What is the definition of a dative covalent bond?
  A The complete transfer of valence electrons from one atom to another
  B When a pair of electrons are shared between two atoms, each having donated an electron
  C When both electrons in a bond have been donated by the same atom
  D An electrostatic attraction between two atoms
  E The attraction between delocalised electrons and positively charged ions
21. Which of the following affects the brain?
  A Muscular dystrophy
  B Multiple Sclerosis
  C Cardiovascular Disease
  D Type II Diabetes
  E Hodgkin Lymphoma
22. Who wrote ‘The Origin of the Species?’
  A Gregor Mendel
  B Hans Christian Anderson
  C Charles Darwin
  D Isaac Newton
  E Albert Einstein
23. What do plant cells have that animal cells do not?
  A Mitochondria
  B Nucleus
  C Cell wall
  D Cell Membrane
  E Golgi apparatus
24. The Central Nervous System (CNS) is comprised of which of the following?
  A The brain and the heart
  B The heart and the blood vessels
  C The brain and the spinal cord
  D The liver and the pancreas
  E The stomach and the intestines
25. Complete the equation to show the anaerobic respiration of yeast. Glucose → ?
  A Carbon dioxide and water
  B Carbon dioxide and hydrogen
  C Carbon dioxide and oxygen
  D Carbon dioxide and ethanol
  E Carbon dioxide and carbon monoxide
26. Which of the following helps to maintain cell membrane stability?
  A Cholesterol
  B High temperatures
  C Acidic conditions
  D High pressure
  E Rapid diffusion
27. Which organ of the body is primarily responsible for insulin production?
  A Blood
  B Bowel
  C Stomach
  D Pancreas
  E Liver
28. Which of the following structures is integral for temperature regulation in the body?
  A Baroreceptors
  B Hypothalamus
  C The heart
  D Neurones
  E The spinal cord
29. Which theory is used to describe the physical state and properties of solids, liquids and gases?
  A Gas theory
  B Solid theory
  C Liquid theory
  D Physical theory
  E Kinetic theory
30. What type of fluid is found in the Ventricular System?
  A Blood
  B Cerebrospinal Fluid
  C Lymph
  D Semen
  E Urine
31. Which of the following theories is thought to most accurately describe the way in which an enzyme and substrate fit together?
  A Evolution
  B The induced fit hypothesis
  C The theory of relativity
  D Quantum theory
  E Chemical theory
32. Identify which of the following disorders applies exclusively to women who have given birth (recently).
  A Unipolar depression
  B Bipolar disorder
  C Fluctuating hormone levels
  D Postpartum depression
  E Narcissistic personality disorder
33. Which of the following is not an anxiety disorder?
  A Alzheimer’s disease
  B Obsessive compulsive disorder
  C Panic disorder
  D Post-traumatic stress disorder
  E Agoraphobia
34. Paranoia is a common symptom of which of the following disorders?
  A Unipolar depression
  B Schizophrenia
  C Agoraphobia
  D Epilepsy
  E Multiple sclerosis
35. Which of the following is the chemical formula for table salt?
  A MgCl2
  B NaCl
  C NaCl2
  D KCl
  E BeCl2
36. Which of the following is an aromatic hydrocarbon?
  A Ethene
  B Ethane
  C Benzene
  D Hexane
  E Octene
37. Which of the following is a respiratory disease?
  A Chronic obstructive pulmonary disorder
  B Hypertension
  C Parkinson’s Disease
  D Polio
  E Alzheimer’s disease
38. Which of the following is often prescribed to patients with high cholesterol levels?
  A Beta blocker
  B Non-steroidal anti-inflammatory drugs
  C Statins
  D Opioids
  E Paracetamol
39. Where is the heart located?
  A In the abdomen
  B On the right-hand side of the thoracic cavity
  C On the left-hand side of the thoracic cavity
  D In the chest
  E In the skull
40. Who is known as the father of genetics?
  A Gregor Mendel
  B Albert Einstein
  C Gustav Klimt
  D Charles Darwin
  E Alexander Fleming
